# Indoor MIMO-VLC Using Angle Diversity Transmitters

**DOI:** 10.3390/s22145436

**Published:** 2022-07-21

**Authors:** Biao Qin, Wanli Wen, Min Liu, Yanchao Zhang, Chen Chen

**Affiliations:** 1 School of Microelectronics and Communication Engineering, Chongqing University, Chongqing 400044, China; 202012131131@cqu.edu.cn (B.Q.); liumin@cqu.edu.cn (M.L.); c.chen@cqu.edu.cn (C.C.); 2 Information Optoelectronics Research Institute, Harbin Institute of Technology at Weihai, Weihai 264209, China; yanchaozhang@hit.edu.cn

**Keywords:** visible light communication (VLC), multiple-input multiple-output (MIMO), angle diversity transmitter (ADT)

## Abstract

In this paper, we, for the first time, apply angle diversity transmitters (ADTs) to enhance the performance of multiple-input multiple-output visible light communication (MIMO-VLC) systems. The ADT is designed to consist of one center light emitting diode (LED) and multiple inclined side LEDs. We calculate the line-of-sight (LOS) channel gain of the MIMO-VLC system using ADTs and further derive the average achievable rate of the system. We show that the average achievable rate is related to both the inclination angle of the side LEDs and the spacing between two adjacent ADTs in the MIMO-VLC system. Simulations are conducted to verify that the average achievable rate of the ADT-enhanced MIMO-VLC system can be maximized by setting the optimal inclination angle of the side LEDs and the optimal spacing between adjacent ADTs. The obtained results further show that the average achievable rate of the ADT-enhanced MIMO-VLC system can be greatly improved when there are more LEDs in each ADT. Specifically, a substantial 42.9% average achievable rate improvement can be achieved by using the optimized ADT in comparison to using a conventional non-angle diversity transmitter.

## 1. Introduction

Nearly two-thirds of the world’s population will have internet access by 2023, with a total of 5.3 billion users and 29.3 billion connected devices [1], which brings a great challenge to the radio frequency (RF) technology facing the spectrum crisis. In order to solve this problem, increasingly researchers turn their attention to the visible light communication (VLC) technology. As a supplement to RF technology, VLC can use visible light with a wavelength of 380 to 780 nm as an information transmission carrier, and the abundant spectrum resources can effectively solve the spectrum crisis [2]. VLC uses light emitting diodes (LEDs) for signal transmission and photodiodes (PDs) for signal detection [3]. Compared with traditional RF technologies, VLC has the advantages of no electromagnetic radiation, high security and no spectrum licensing requirement [4], which has many emerging applications, such as indoor precise positioning, underwater communication and military applications [5,6,7].

In indoor environments, multiple LEDs that provide lighting are usually fixed on the ceiling, and the LEDs are able to act as transmitters to send signals to users, which makes it possible to build an indoor multiple-input multiple-output VLC (MIMO-VLC) system [8]. Owing to its ability to provide substantial multiplexing gain and, hence, enhance the system capacity, MIMO-VLC has attracted great research interest in recent years [9,10,11]. Nevertheless, due to the line-of-sight (LOS) transmission and the small spacing of PDs, practical MIMO-VLC systems suffer from high channel correlation, and hence the achievable multiplexing gain is not significant.

In order to enhance the performance of MIMO-VLC systems, many emerging techniques have been considered for MIMO-VLC systems, such as hybrid space–frequency domain pre-equalization [12], two-dimensional power allocation [13], user-centric MIMO techniques [14], generalized MIMO techniques [15], advanced multiple access techniques [16,17] and angle diversity receiver (ADR)-based advanced MIMO reception techniques [18,19,20,21].

The use of ADR with a proper diversity combining scheme in MIMO-VLC systems can reduce the channel correlation and, hence, enhance the system performance. Similarly, angle diversity can also be introduced at the transmitter side by an angle diversity transmitter (ADT) for VLC systems. Thus far, ADT has been applied in various VLC systems in the literature. In [22], ADT was applied in visible light positioning (VLP) systems to enhance the robustness against accelerometer measurement errors.

In [23], ADT was used to realize space division multiple access (SDMA) in optical attocell network, which was shown to achieve improved area spectrum efficiency compared with traditional time division multiple access (TDMA). In [24], ADT was adopted to improve the bit error rate (BER) performance of indoor single-cell systems. However, ADT has not yet been considered in MIMO-VLC systems, and whether the use of ADT can enhance the performance of MIMO-VLC systems has not been verified.

In this paper, for the first time, to the best of our knowledge, we introduce ADT into MIMO-VLC systems, and the designed ADT includes one center LED and multiple inclined side LEDs. The line-of-sight (LOS) channel gain of the MIMO-VLC system using ADTs is calculated, and the average achievable rate of the ADT-enhanced MIMO-VLC system is further derived. We found that the average achievable rate of the ADT-enhanced MIMO-VLC system can be maximized by setting the optimal inclination angle of the side LEDs and the optimal spacing between adjacent ADTs. Simulations are performed to verify the superiority of applying ADTs in MIMO-VLC systems. The impact of the transmitted SNR and the PD spacing is also analyzed.

## 2. System Model

**Figure 1:**
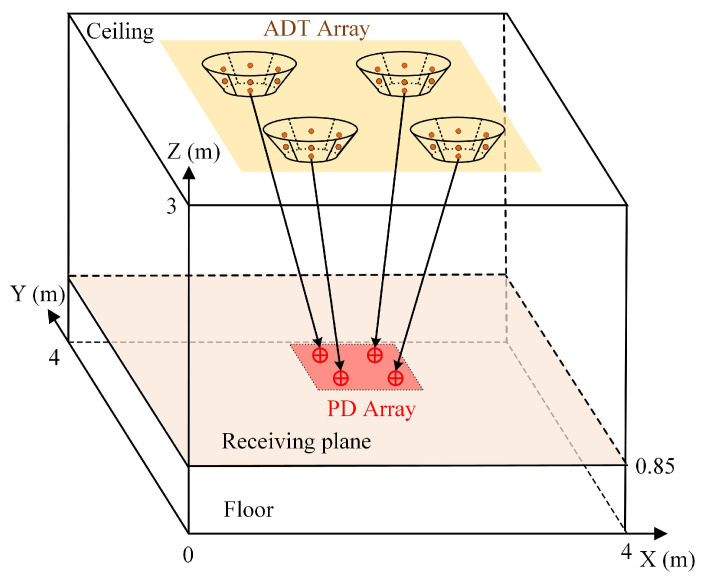
Geometric setup of a general MIMO-VLC system.

In this section, we consider a general indoor MIMO-VLC system model, and its geometric setup is illustrated in Figure 1. An ADT array consisting of $N_{t}$ ADTs are installed on the ceiling and used as transmitters to transmit data. The PD array composed of $N_{r}$ PDs is located at the user plane. Usually, the light received by the PD consists of two parts: one is the LOS component, and the other is the non-line-of-sight (NLOS) component. Previous studies have shown that the power of the LOS component is usually much larger than that of the NLOS component [9]. Therefore, we only consider the influence of LOS component on the MIMO-VLC system performance in the following.

Assuming that each LED follows the Lambertian radiation pattern, the LOS channel gain from LED to PD can be expressed as follows [25]:

(1) $$h=\frac{\left(\mu +1\right)S_{d}}{2\pi L^{2}}\cos^{\mu }\left(\alpha \right)\delta \tau cos\left(\beta \right),$$

where μ=−ln2/ln(cos(Ψ)) is the Lambertian emission order and Ψ is the semi-angle at half power of the LED; $S_{d}$ is the active area of the PD; *L* is the distance between LED and PD; the emission angle and the incident angle are expressed by α and β, respectively; δ is the gain of optical filter; and τ is the gain of lens. Note that *h* will be zero when the incident angle is larger than the FOV of the PD or the emission angle is larger than the semi-angle of the LED.

For a typical MIMO-VLC system equipped with $N_{t}$ LED and $N_{r}$ PDs, letting x = ${\left[x_{1},x_{2},\cdots ,x_{N_{t}}\right]}^{T}$ denote the transmitted signal vector, H represent the $N_{r}\times N_{t}$ MIMO channel matrix and w = [$w_{1},w_{2},\cdots ,w_{N_{r}}{]}^{T}$ be the additive noise vector, the received signal vector y = ${\left[y_{1},y_{2},\cdots ,y_{N_{r}}\right]}^{T}$ can be expressed by

(2) y=Hx+w,

where the channel matrix of $N_{r}\times N_{t}$ MIMO-VLC system can be given by

(3) $$H=\left[\begin{array}{ccc} h_{11} & \cdots & h_{1N_{t}}\\ \vdots & \ddots & \vdots \\ h_{N_{r}1} & \cdots & h_{N_{r}N_{t}} \end{array}\right],$$

where $h_{im}$$\left(i=1,2,...,N_{r};m=1,2,...,N_{t}\right)$ denotes the channel gain between the *i*-th PD and the *m*-th LED.

It can be seen from (2) that the output signal from each PD is a function of the input signal $x_{m}$ and the additive noise $w_{i}$. In order to recover the transmitted signal at the receiver side, zero-forcing (ZF) based MIMO demultiplexing is adopted because of its simplicity and low computational complexity. Assuming H is full-column-rank ($N_{r}\geq N_{t}$), the transmitted signal vector x can be obtained by

(4) $$\hat{x}=\tilde{H}y=x+\tilde{H}w,$$

where $\tilde{H}$ denotes the pseudo inverse of H, which can be calculated by

(5) $$\tilde{H}={\left(H^{*}H\right)}^{-1}H^{*},$$

where $H^{*}$ denotes the conjugated transpose of H.

The additive noise includes thermal noise and shot noise [25], which can be modeled as a real-valued additive white Gaussian noise (AWGN). The noise power can be calculated by $P_{w}=N_{0}B$, where $N_{0}$ and *B* denote the noise power spectral density (PSD) and the signal bandwidth, respectively.

## 3. MIMO-VLC Using ADTs

In this section, we present the principle of MIMO-VLC systems using ADTs. First, the structure of ADT is introduced, then the LOS channel gain using ADT is derived, and finally the average achievable rate of MIMO-VLC systems using ADTs is obtained.

### 3.1. Structure of ADT

**Figure 2:**
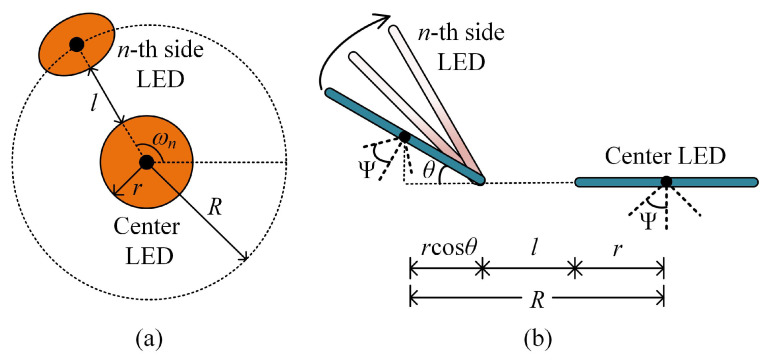
(**a**) Top view and (**b**) side view of the ADT. Only the center LED and the *n*-th side LED are shown for illustration.

We assume that each ADT consists of totally *N* LEDs, including one center LED and N−1 inclined side LEDs. More specifically, the center LED is placed horizontally, while the N−1 side LEDs are inclined with the same inclination angle. It is further assumed that all the LEDs in the ADT have the same optical and electrical performance and the same radius *r*. The top view and the side view of the designed ADT are illustrated in Figure 2a,b, respectively. It can be seen from Figure 2a that the side LEDs are placed at the circumference of a circle with radius *R*, while the center LED is located at the center of the circle. Moreover, the gap between the center LED and the side LED is *l*, and the azimuth angle and the inclination angle of the *n*-th side LED are $\omega_{n}$ and θ, respectively. The azimuth angle $\omega_{n}$ can be given as follows:

(6) $$\omega_{n}=\frac{2\pi (n-1)}{N-1},n=1,2,\ldots ,N-1.$$

For example, when there are four LEDs (N=4) in the ADT, the corresponding azimuth angles of the three side LEDs are $0^{\circ }$, $120^{\circ }$, $240^{\circ }$, respectively.

### 3.2. LOS Channel Gain Using ADT

**Figure 3:**
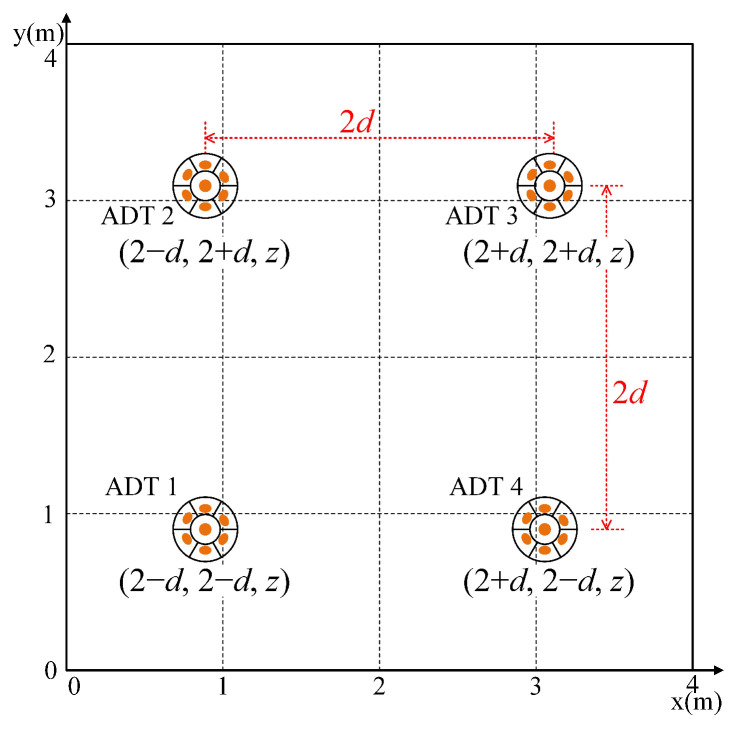
Placement of four ADTs in the ceiling.

Figure 3 illustrates the placement of four ADTs in the ceiling by considering an indoor $N_{r}\times N_{t}$ MIMO-VLC system with $N_{t}=4$, where the coordinates of the four center LEDs in the four ADTs are (2−d,2−d,z), (2−d,2+d,z), (2+d,2+d,z) and (2+d,2−d,z) with unit meter. Hence, the spacing between two adjacent ADTs is 2d. As the coordinates of the center LED in each ADT are known, the LOS channel gain of the center LED in the *m*-th ($m=1,2,\ldots ,N_{t}$) ADT to the *i*-th ($i=1,2,\ldots ,N_{r}$) PD, i.e., $h_{i,m0}$, can be calculated by using (1).

However, the LOS channel gains of the side LEDs in each ADT to each PD cannot be calculated directly utilizing (1) due to the distinctive positions and orientations of the side LEDs in the same ADT. In order to calculate the LOS channel gains of the side LEDs in the ADT, the coordinates and emission angle of each side LED should be determined first.

Let $\left(x_{m0},y_{m0},z_{m0}\right)$ and $\left(x_{mn},y_{mn},z_{mn}\right)$ denote the coordinates of positions of the center LED and the *n*-th (n=1,2,…,N−1) side LED in the *m*-th ADT, respectively. Given the azimuth angle $\omega_{n}$ and the inclination angle θ of the *n*-th side LED, according to Figure 2b, the coordinates of the *n*-th side LED in the *m*-th ADT can be obtained as follows:

(7) $$\left\{\begin{array}{c} x_{mn}=x_{m0}+\left[l+r\left(1+\cos\theta \right)\right]\cos\omega_{n}\\ y_{mn}=y_{m0}+\left[l+r\left(1+\cos\theta \right)\right]\sin\omega_{n}\\ z_{mn}=z_{m0}+r\sin\theta \end{array}\right..$$

**Figure 4:**
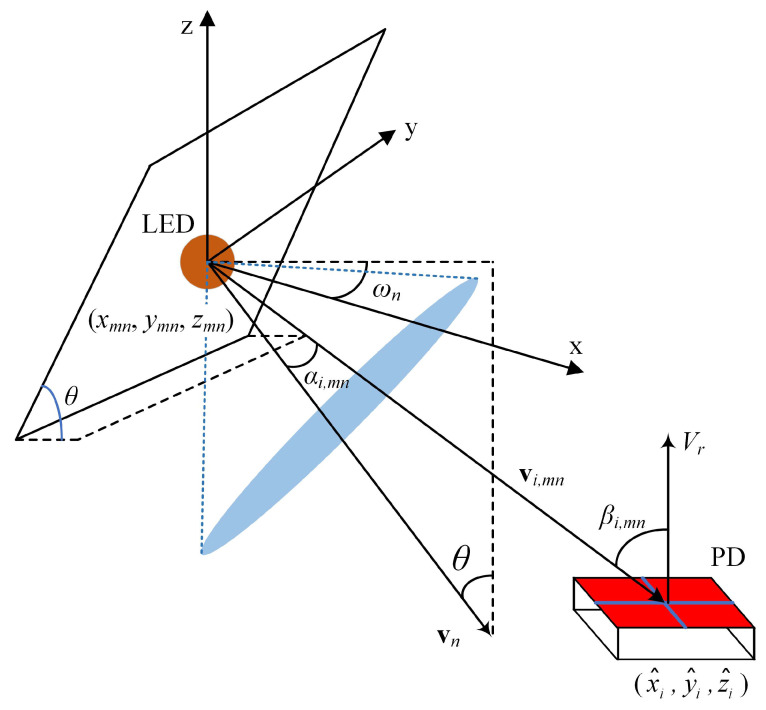
Geometry for LOS channel gain calculation of the side LEDs in the ADT.

Figure 4 shows the geometry for the calculation of the LOS channel gain of the side LEDs in the ADT, where the positions of the *n*-th side LED in the *m*-th ADT and the *i*-th PD are $\left(x_{mn},y_{mn},z_{mn}\right)$ and $\left({\hat{x}}_{i},{\hat{y}}_{i},{\hat{z}}_{i}\right)$, respectively. Hence, the corresponding incident angle $\beta_{i,mn}$ can be given by

(8) $$\cos\beta_{i,mn}=\frac{z_{mn}-{\hat{z}}_{i}}{\sqrt{{\left({\hat{x}}_{i}-x_{mn}\right)}^{2}+{\left({\hat{y}}_{i}-y_{mn}\right)}^{2}+{\left({\hat{z}}_{i}-z_{mn}\right)}^{2}}}.$$

As the azimuth angle $\omega_{n}$ and the inclination angle θ of the side LED change, the emission angle $\alpha_{i,mn}$ from the *n*-th side LED in the *m*-th ADT to the *i*-th PD changes accordingly. Let $v_{n}$ denote the normal vector of the plane of the side LED and $v_{i,mn}$ represent the vector from the side LED to the PD; hence, we have:

(9) $$\cos\alpha_{i,mn}=\frac{\left(v_{i,mn},v_{n}\right)}{\left|\right|v_{i,mn}\left|\right|\cdot \left|\right|v_{n}\left|\right|},$$

where ||·|| and $\left(\cdot ,\cdot \right)$ denote the norm of vector and inner product of two vectors, respectively.

Given the azimuth angle $\omega_{n}$ and the inclination angle θ of the side LED, the vector $v_{n}$ of the plane of the side LED can be calculated by

(10) $$v_{n}=\left(\sin\theta \cos\omega_{n},\sin\theta \sin\omega_{n},-\cos\theta \right).$$

Moreover, given the positions of the *n*-th side LED in the *m*-th ADT and the *i*-th PD, the vector $v_{i,mn}$ is obtained by

(11) $$v_{i,mn}=\left({\hat{x}}_{i}-x_{mn},{\hat{y}}_{i}-y_{mn},{\hat{z}}_{i}-z_{mn}\right).$$

Substituting (10) and (11) into (9), we have:

(12) $$\begin{array}{cc} \; & \cos\alpha_{i,mn}=\\ \; & \frac{\left[\left({\hat{x}}_{i}-x_{mn}\right)\cos\omega_{n}+\left({\hat{y}}_{i}-y_{mn}\right)\sin\omega_{n}\right]\sin\theta -\left({\hat{z}}_{i}-z_{mn}\right)\cos\theta }{{\left[{\left({\hat{x}}_{i}-x_{mn}\right)}^{2}+{\left({\hat{y}}_{i}-y_{mn}\right)}^{2}+{\left({\hat{z}}_{i}-z_{mn}\right)}^{2}\right]}^{1/2}}. \end{array}$$

Finally, the LOS channel gain between the *n*-th side LED in the *m*-th ADT and the *i*-th PD, i.e., $h_{i,mn}$ (n=1,…,N−1), can be successfully calculated by substituting (8) and (12) into (1). Since all the LEDs in each ADT transmit the same signal, the overall LOS channel gain between the *m*-th ADT and the *i*-th PD is contributed by both the center LED and the N−1 side LEDs, which can be given as follows:

(13) $$h_{i,m}=h_{i,m0}+\sum_{n=1}^{N-1}h_{i,mn}.$$

### 3.3. Average Achievable Rate

In this work, spatial multiplexing (SMP)-based MIMO-VLC is considered and, hence, different ADTs transmit different data streams simultaneously [10]. According to (2), (3) and (13), the *m*-th data stream at the *k*-th receiver location in the $N_{r}\times N_{t}$ MIMO-VLC system can be expressed by

(14) $${\hat{x}}_{m}^{k}=x_{m}^{k}+\sum_{i=1}^{N_{r}}{\tilde{h}}_{i,m}^{k}w_{i},$$

where ${\tilde{h}}_{i,m}^{k}$ denotes the element in the *i*-th row and *m*-column of ${\tilde{H}}^{k}$ with $H^{k}$ being the corresponding $N_{r}\times N_{t}$ MIMO channel matrix at the *k*-th receiver location.

Based on (14), the signal-to-noise ratio (SNR) of the estimate of the *m*-th data stream at the *k*-th receiver location can be obtained by

(15) $$\Gamma_{m}^{k}=\frac{P_{s}}{\sum_{i=1}^{N_{r}}{\left({\tilde{h}}_{i,m}^{k}\right)}^{2}P_{w}}=\frac{\Gamma_{0}}{\sum_{i=1}^{N_{r}}{\left({\tilde{h}}_{i,m}^{k}\right)}^{2}},$$

where $P_{s}$ is the electrical power of the transmitted signal, $\Gamma_{0}$ is the transmitted SNR, which is defined as $\Gamma_{0}=\frac{P_{s}}{P_{w}}$ [9].

As a result, the achievable rate (bits/s/Hz) of the *m*-th data stream at the *k*-th receiver location is calculated by [14]

(16) $$R_{m}^{k}=\frac{1}{2}\log_{2}\left(1+\Gamma_{k,m}\right)=\frac{1}{2}\log_{2}\left(1+\frac{\Gamma_{0}}{\sum_{i=1}^{N_{r}}{\left({\tilde{h}}_{i,m}^{k}\right)}^{2}}\right).$$

Hence, the overall achievable rate of the ADT-based $N_{r}\times N_{t}$ MIMO-VLC system at the *k*-th receiver location can be achieved as follows’:

(17) $$\begin{array}{c} R^{k}=\sum_{m=1}^{N_{t}}R_{m}^{k}=\frac{1}{2}\sum_{m=1}^{N_{t}}\log_{2}\left(1+\frac{\Gamma_{0}}{\sum_{i=1}^{N_{r}}{\left({\tilde{h}}_{i,m}^{k}\right)}^{2}}\right), \end{array}$$

and the average achievable rate at *K* different receiver locations over the receiving plane, with k=1,2,⋯,K, can be given by

(18) $$R=\frac{\sum_{k=1}^{K}R_{k}}{K}=\frac{1}{2K}\sum_{k=1}^{K}\sum_{m=1}^{N_{t}}\log_{2}\left(1+\frac{\Gamma_{0}}{\sum_{i=1}^{N_{r}}{\left({\tilde{h}}_{i,m}^{k}\right)}^{2}}\right).$$

By observing (18), we find that the average achievable rate of the ADT-based $N_{r}\times N_{t}$ MIMO-VLC system is related by the term ${\tilde{h}}_{i,m}^{k}$, which is largely determined by the structure and the spacing of the ADTs. Therefore, it is feasible to optimize the structure (e.g., the inclination angle of the side LEDs) and the spacing of the ADTs to maximize the average achievable rate of the system.

## 4. Simulation Results

In this section, we evaluate the average achievable rate of a typical indoor 4 × 4 ($N_{r}$ = $N_{t}$ = 4) MIMO-VLC system applying the designed ADTs. As depicted in Figure 1 and Figure 3, the room is assumed to have a dimension of 4 × 4 × 3 m, and the square ADT array is centered at the ceiling of the room.

The height of the receiving plane is 0.85 m, and a total of *K* = 40,000 uniformly distributed receiver locations are considered to calculate the average achievable rate using (18). Moreover, the center LED of each ADT is assumed to have a large semi-angle at half power of $70^{\circ }$ to guarantee the full coverage of the receiving plane, while the semi-angle at half power of all the side LEDs is set to $30^{\circ }$. The key simulation parameters can be found in Table 1.

**Figure 5:**
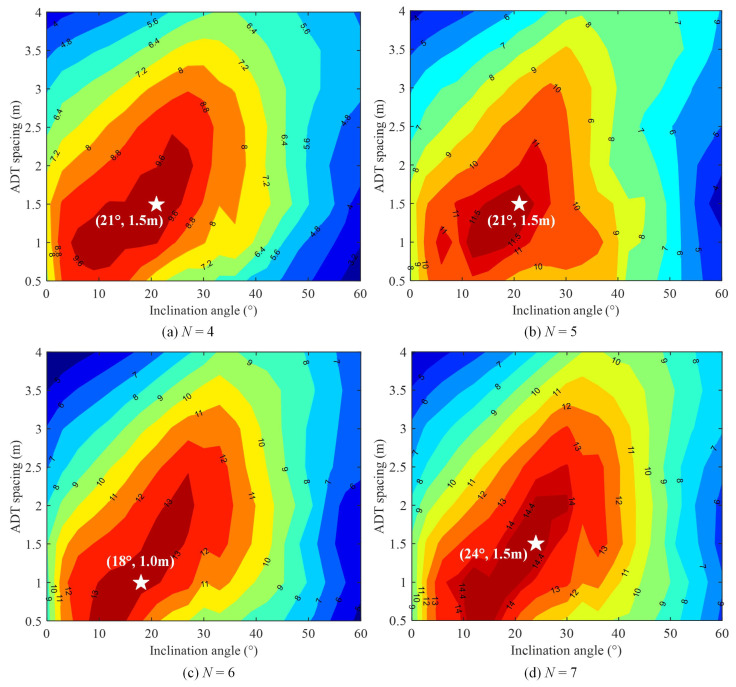
Contour plot of the average achievable rate (bit/s/Hz) vs. inclination angle θ and ADT spacing 2d for (**a**) N=4, (**b**) N=5, (**c**) N=6 and (**d**) N=7.

First, we evaluate the average achievable rate with respect to the inclination angle θ and the ADT spacing 2d for the ADTs each consisting of *N* LEDs. By setting $\theta \in [0^{\circ },60^{\circ }]$ with a step of $3^{\circ }$ and 2d∈ [0.5 m, 4 m] with a step of 0.5 m, we aim to obtain an optimal combination of θ and 2d that can maximize the average achievable rate of the 4 × 4 MIMO-VLC system for a given *N* value. Figure 5a–d show the contours of the average achievable rate versus the inclination angle θ and the ADT spacing 2d with a transmitted SNR of 150 dB for *N* = 4, 5, 6 and 7, respectively. As we can see, the optimal combination of θ and 2d to achieve the maximum average achievable rate for each case is highlighted in the white star in Figure 5.

For the case of *N* = 4, the maximum average achievable rate of about 10.1 bit/s/Hz is obtained with an optimal inclination angle of θ = $21^{\circ }$ and an optimal ADT spacing of 2d = 1.5 m. Similarly, for the case of *N* = 5, the maximum average achievable rate of about 12.0 bit/s/Hz is achieved with the same optimal combination of θ = $21^{\circ }$ and 2d = 1.5 m. However, for the case of *N* = 6, the optimal combination of θ and 2d is given by θ = $18^{\circ }$ and 2d = 1.0 m, and the corresponding maximum average achievable rate is increased to 13.4 bits/s/Hz.

Moreover, for the case of *N* = 7, the maximum average achievable rate is about 15.2 bits/s/Hz, which is achieved with an optimal inclination angle of θ = $24^{\circ }$ and an optimal ADT spacing of 2d = 1.5 m. It can be concluded from Figure 5 that it is feasible and beneficial to optimize both the inclination angle θ and the ADT spacing 2d to maximize the average achievable rate of the 4 × 4 MIMO-VLC system applying ADTs. Moreover, the maximum average achievable rate of the system is gradually increased with an increased number of LEDs in each ADT.

**Figure 6:**
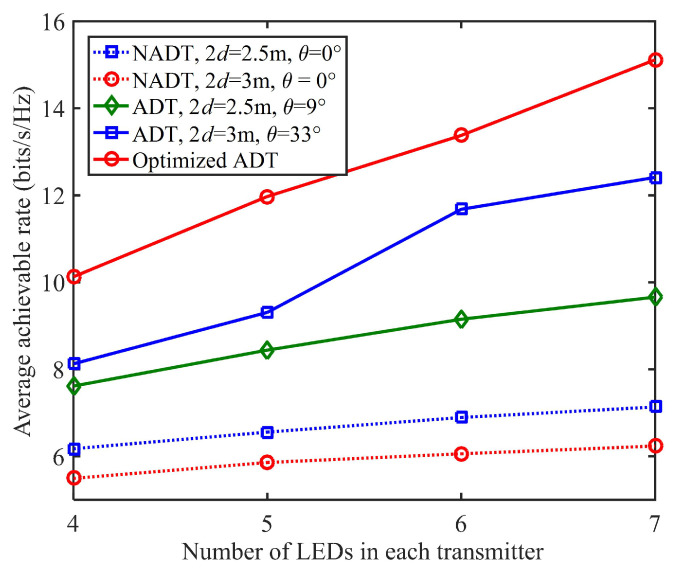
Average achievable rate vs. the number of LEDs in each transmitter.

Next, we compare the average achievable rate of the 4 × 4 MIMO-VLC system using different types of transmitters, including (1) conventional non-angle diversity transmitter (NADT) with 2d = 2.5 m/3 m and θ = $0^{\circ }$, (2) non-optimized ADT with 2d = 2.5 m/3 m and θ = $9^{\circ }$/$33^{\circ }$ and (3) optimized ADT with the optimal combination of θ and 2d. Figure 6 shows the average achievable rate versus the number of LEDs in each transmitter for different types of transmitters, where the transmitted SNR is 150 dB.

As we can see, the average achievable rates are all gradually increased when there are more LEDs in each transmitter for all types of transmitters, which suggests the advantage to design the transmitter with more LEDs under the conditions of increased complexity and cost. Particularly, the improvement of average achievable rate is much more significant for the optimized ADT in comparison to the conventional NADT when increasing the number of LEDs in each transmitter. For example, the average achievable rate using NADT with 2d = 2.5 m, and θ = $0^{\circ }$ is improved from 6.2 to 7.1 bits/s/Hz when the number of LEDs in each transmitter is increased from *N* = 4 to 7, which corresponds to an average achievable rate improvement of about 14.5%.

In contrast, a significant 49.5% average achievable rate improvement can be achieved by using the optimized ADT, when the number of LEDs in each transmitter is increased from *N* = 4 to 7. Moreover, for the same number of LEDs in each transmitter, the 4 × 4 MIMO-VLC system using ADT can generally obtain a higher average achievable rate than that using conventional NADT, while the 4 × 4 MIMO-VLC system using optimized ADT can always achieve the highest average achievable rate.

More specifically, for the case that each transmitter consists of *N* = five LEDs, the average achievable rates using NADT with 2d = 2.5 m and θ = $0^{\circ }$, non-optimized ADT with 2d = 2.5 m and θ = $9^{\circ }$ and optimized ADT are 6.6, 8.4 and 12.0 bits/s/Hz, respectively. Hence, an average achievable rate improvement of 27.3% is obtained by the non-optimized ADT with 2d = 2.5 m and θ = $9^{\circ }$ in comparison to NADT with 2d = 2.5 m and θ = $0^{\circ }$, while the optimized ADT outperforms the NADT with 2d = 2.5 m and θ = $0^{\circ }$ by an average achievable rate improvement of up to 42.9%.

**Figure 7:**
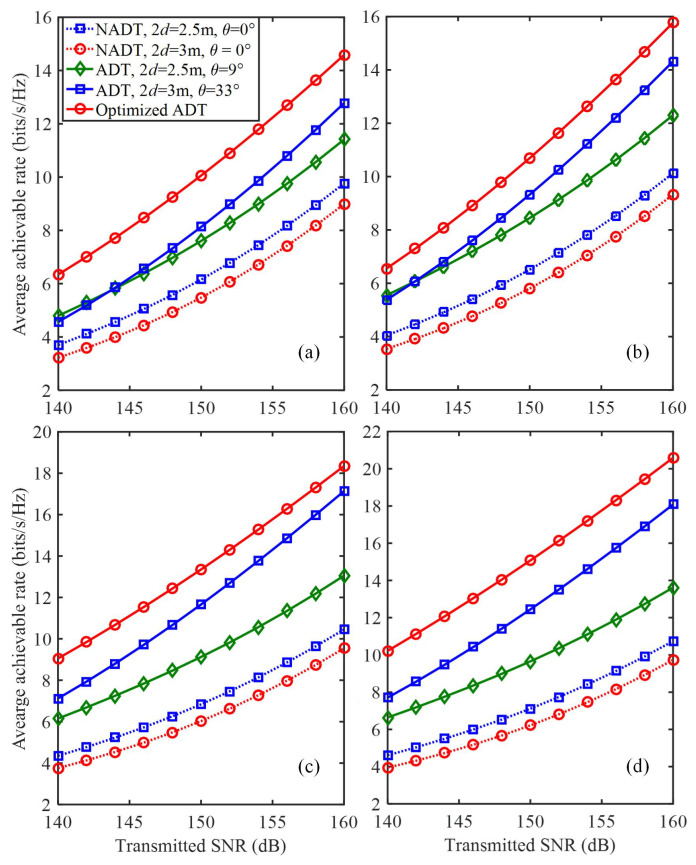
Average achievable rate vs. the transmit SNR in the generalized ADT with different *N* values for (**a**) *N* = 4, (**b**) *N* = 5, (**c**) *N* = 6 and (**d**) *N* = 7.

We further investigate the impact of transmitted SNR and the PD spacing on the average achievable rate of the 4 × 4 MIMO-VLC system using different types of transmitters. Figure 7a–d shows the average achievable rate versus the transmitted SNR using different types of transmitters for *N* = 4, 5, 6 and 7, respectively. It can be clearly observed that the average achievable rates are all gradually increased with the increase of the transmitted SNR for all types of transmitters. Moreover, to reach the same average achievable rate, the 4 × 4 MIMO-VLC system using optimized ADT always requires the smallest transmitted SNR in comparison to other benchmark schemes.

For example, for the case of *N* = 5, the required transmitted SNRs to reach an average achievable rate of 8 bits/s/Hz for NADT with 2d = 2.5 m and θ = $0^{\circ }$, non-optimized ADT with 2d = 2.5 m and θ = $9^{\circ }$ and optimized ADT are about 155, 149 and 144 dB, respectively. Hence, transmitted SNR reductions of 11 and 5 dB are obtained by using optimized ADT in comparison to that using NADT with 2d = 2.5 m and θ = $0^{\circ }$ and non-optimized ADT with 2d = 2.5 m, respectively.

**Figure 8:**
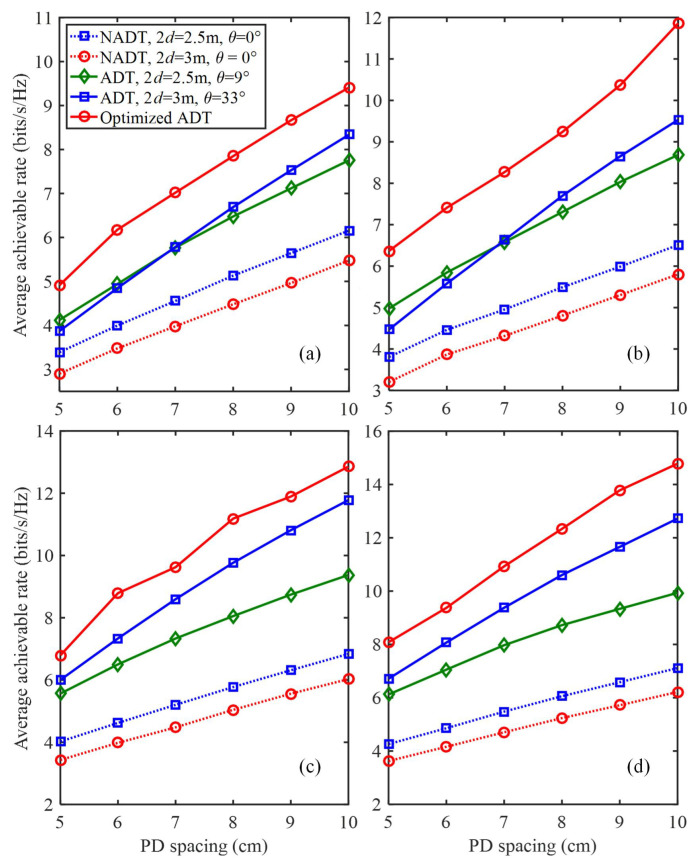
Average achievable rate vs. the spacing between two adjacent PDs in the generalized ADT with different *N* values for (**a**) *N* = 4, (**b**) *N* = 5, (**c**) *N* = 6 and (**d**) *N* = 7.

Figure 8a–d shows the average achievable rate versus the PD spacing using different types of transmitters for *N* = 4, 5, 6 and 7, respectively. Similarly, we found that the average achievable rates are all gradually increased with the increase of the PD spacing for all types of transmitters, and the 4 × 4 MIMO-VLC system using optimized ADT always requires the smallest transmitted SNR to reach the same average achievable rate in comparison to other benchmark schemes.

## 5. Discussions on Practical Implementation of ADTs in MIMO-VLC Systems

In this section, we discuss the practical implementation issues of applying ADTs in indoor MIMO-VLC systems. Considering the low emitted optical power of a single LED chip, a practical LED lamp usually consists of an array of LED chips, which naturally has an array structure [25]. Generally, a planar array structure is adopted in practical LED lamps due to its simplicity and low complexity. As a result, an ADT can be achieved by modifying the planar array structure of a practical LED lamp.

More specifically, we only need to purposely tilt some of the LED chips in the array with the pre-determined tilting angles, as illustrated in Figure 2, and then a planar array can be transformed into an ADT. Differing from conventional planar LED arrays, which usually have a two-dimensional (2D) structure, the ADTs might have a three-dimensional (3D) structure. Hence, the implementation complexity and cost of an ADT are generally higher than those of a conventional LED lamp with a planar LED array. Despite the difference in the array structure, ADTs can adopt the same directional LED chips and the same driving circuit as those used in conventional LED lamps.

## 6. Conclusions

In this paper, we proposed and evaluated novel ADT-enhanced MIMO-VLC systems, where the ADT was designed to consist of one center LED and multiple inclined side LEDs. The LOS channel gain using ADT was calculated, and the average achievable rate of the MIMO-VLC system applying ADTs was further derived. Our simulation results verified that the average achievable rate of the ADT-enhanced MIMO-VLC system can be maximized by selecting the optimal combination of the inclination angle of the side LEDs and the spacing between adjacent ADTs.

We also demonstrated that the average achievable rate of the ADT-enhanced MIMO-VLC system can be further enhanced by equipping the ADT with more LEDs under the conditions of increased complexity and cost. Therefore, it is promising to apply optimized ADTs for substantial performance enhancement of high-speed MIMO-VLC systems.

## Figures and Tables

**Table 1:** Simulation parameters.

Parameter	Value
Room dimension	4 m × 4 m × 3 m
Height of receiving plane	0.85 m
PD spacing	10 cm
Semi-angle at half power of center LED	$70^{\circ }$
Semi-angle at half power of side LED	$30^{\circ }$
Half-angle FOV photodetector	$70^{\circ }$
Gain of optical filter	1
Refractive index of optical lens	1.5
Responsivity of the PD	1 A/W
Radius of LED	0.5 cm
Gap between center and side LED	5 cm
Active area of PD	1 ${\mathrm{cm}}^{2}$
Signal bandwidth	10 MHz
Noise PSD	$10^{-22}A^{2}/\mathrm{Hz}$

## Data Availability

Not applicable.

## References

[1] Cisco U. (2020). Cisco Annual Internet Report (2018–2023) White Paper.

[2] Le Minh H., Ghassemlooy Z., O’Brien D., Faulkner G. Indoor gigabit optical wireless communications: Challenges and possibilities. Proceedings of the 12th International Conference on Transparent Optical Networks (ICTON).

[3] Grubor J., Randel S., Langer K.D., Walewski J.W. (2008). Broadband information broadcasting using LED-based interior lighting. J. Lightw. Technol..

[4] Hussain S.I., Abdallah M.M., Qaraqe K.A. Hybrid radio-visible light downlink performance in RF sensitive indoor environments. Proceedings of the sixth International Symposium on Communications, Control and Signal Processing (ISCCSP).

[5] Armstrong J., Sekercioglu Y.A., Neild A. (2013). Visible light positioning: A roadmap for international standardization. IEEE Commun. Mag..

[6] Pathak P.H., Feng X., Hu P., Mohapatra P. (2015). Visible light communication, networking, and sensing: A survey, potential and challenges. IEEE Commun. Surv. Tuts..

[7] Wu D., Zhong W.D., Ghassemlooy Z., Chen C. (2016). Short-range visible light ranging and detecting system using illumination light emitting diodes. IET Optoelectron..

[8] Zeng L., O’Brien D.C., Le Minh H., Faulkner G.E., Lee K., Jung D., Oh Y., Won E.T. (2009). High data rate multiple input multiple output (MIMO) optical wireless communications using white LED lighting. IEEE J. Sel. Areas Commun..

[9] Fath T., Haas H. (2013). Performance comparison of MIMO techniques for optical wireless communications in indoor environments. IEEE Trans. Commun..

[10] Lian J., Brandt-Pearce M. (2017). Multiuser MIMO indoor visible light communication system using spatial multiplexing. J. Lightw. Technol..

[11] Chen C., Zhong W.D., Wu D. (2017). On the coverage of multiple-input multiple-output visible light communications [Invited]. J. Opt. Commun. Netw..

[12] Chen C., Zhong W.D. (2017). Hybrid space-frequency domain pre-equalization for DC-biased optical orthogonal frequency division multiplexing based imaging multiple-input multiple-output visible light communication systems. Opt. Eng..

[13] Deng X., Fan W., Cunha T.B., Ma S., Chen C., Dong Y., Zou X., Yan L., Linnartz J.P. (2022). Two-dimensional power allocation for optical MIMO-OFDM systems over low-pass channels. IEEE Trans. Veh. Technol..

[14] Chen C., Yang H., Du P., Zhong W.D., Alphones A., Yang Y., Deng X. (2020). User-centric MIMO techniques for indoor visible light communication systems. IEEE Syst. J..

[15] Chen C., Zhong X., Fu S., Jian X., Liu M., Yang H., Alphones A., Fu H.Y. (2021). OFDM-based generalized optical MIMO. J. Lightw. Technol..

[16] Tsiropoulou E.E., Gialagkolidis I., Vamvakas P., Papavassiliou S. Resource allocation in visible light communication networks: NOMA vs OFDMA transmission techniques. Proceedings of the International Conference on Ad-Hoc Networks and Wireless (ADHOC-NOW).

[17] Chen C., Zhong W.D., Yang H., Du P. (2018). On the performance of MIMO-NOMA-based visible light communication systems. IEEE Photon. Technol. Lett..

[18] Nuwanpriya A., Ho S.W., Chen C.S. (2015). Indoor MIMO visible light communications: Novel angle diversity receivers for mobile users. IEEE J. Sel. Areas Commun..

[19] Mmbaga P.F., Thompson J., Haas H. (2016). Performance analysis of indoor diffuse VLC MIMO channels using angular diversity detectors. J. Lightw. Technol..

[20] Hosney M., Selmy H.A., Srivastava A., Elsayed K.M. (2020). Interference mitigation using angular diversity receiver with efficient channel estimation in MIMO VLC. IEEE Access.

[21] Dixit V., Kumar A. (2021). Performance analysis of angular diversity receiver based MIMO–VLC system for imperfect CSI. J. Opt..

[22] Yin L., Wu X., Haas H. Indoor visible light positioning with angle diversity transmitter. Proceedings of the IEEE 82nd Vehicular Technology Conference (VTC2015-Fall).

[23] Chen Z., Basnayaka D.A., Haas H. (2017). Space division multiple access for optical attocell network using angle diversity transmitters. J. Lightw. Technol..

[24] Dixit V., Kumar A. Performance analysis of indoor visible light communication system with angle diversity transmitter. Proceedings of the IEEE fourth Conference on Information & Communication Technology (CICT).

[25] Komine T., Nakagawa M. (2004). Fundamental analysis for visible-light communication system using LED lights. IEEE Trans. Consum. Electron..

